# Effects of individual and group metacognitive prompts on EFL reading comprehension and incidental vocabulary learning

**DOI:** 10.1371/journal.pone.0215902

**Published:** 2019-05-22

**Authors:** Feng Teng, Barry Lee Reynolds

**Affiliations:** 1 Department of Education Studies, Hong Kong Baptist University, Hong Kong SAR, China; 2 Faculty of Education, University of Macau, Macau SAR, China; Rijksuniversiteit Groningen, NETHERLANDS

## Abstract

Recent research has highlighted the value of providing metacognitive guidance for learning English in a small group setting. This study investigated the effects that the presence or absence of metacognitive prompts for group or individual learning could have on reading comprehension and the incidental learning of vocabulary through reading. A total of 171 university students were randomly assigned to four treatment conditions: collaborative learning with metacognitive prompts, collaborative learning without metacognitive prompts, individual learning with metacognitive prompts, and individual learning without metacognitive prompts. Results indicated that after the treatment, learners in the collaborative learning with metacognitive prompts group outperformed the other groups on both reading comprehension and incidental vocabulary learning assessments. In addition, the vocabulary knowledge acquired by students in the collaborative learning with metacognitive prompts group was highest for meaning recognition, followed by form recognition, meaning recall, and finally form recall. These findings highlight the importance of training students’ self-regulated learning and suggest that the use of metacognitive prompts in a group setting is an effective means to boost EFL reading comprehension and the incidental vocabulary learning for Chinese university students. Pedagogical implications of these and other nuanced findings are discussed.

## Introduction

Second language (L2) vocabulary knowledge is the foundation of learning English as a second (ESL) or a foreign language (EFL) as it has a pronounced effect on language skills such as reading and writing [1]. Out of all the methods of assisting learners in enlarging their second language (L2) vocabulary size, incidental vocabulary learning has been found to be the least robust in the short term but can result in substantial longitudinal vocabulary growth [1, 2]. Incidental vocabulary learning, i.e., picking up new words from reading or listening input without a conscious intention to commit new knowledge to memory [3], has been found to be more challenging compared to intentional vocabulary learning [4]. In addition, previous studies on incidental vocabulary learning have shown partial and arguably unstable gains in vocabulary knowledge [5, 6, 7, 8, 9, 10]. However, the alternative—direct teaching of vocabulary—may be problematic because many teachers may not know where to begin to help learners independently engage in word learning [11]. Moreover, most incidental vocabulary learning studies that show such negligible gains in vocabulary knowledge are those that do not provide learners any instruction other than telling learners to read [12]. While pioneering studies were aimed confirming a hypothesis that incidental learning through reading was possible for L2 learners the same as first language speakers [13, 14]), in recent years the field has begun asking more nuanced pedagogically-oriented critical questions [15].

Vocabulary knowledge is the key to reading comprehension. Without a good mastery of vocabulary knowledge, L2 learners cannot understand what they are reading. Moreover, L2 learners find it challenging to read and reading comprehension has been acknowledged as a major source of the challenge [16, 17].This argument may possibly reflect learners’ need to develop competency in reading as it helps develop content knowledge, extends, consolidates, and sustains vocabulary growth, and creates and sustains motivation to read more. As a result, language teachers recognize the importance of reading and are exploring more effective ways of promoting reading.

Recent studies have suggested that metacognition has an effect on English learners’ vocabulary learning [18, 19]), reading comprehension [17], and writing performance [20, 21]. As argued by Panadero and Alonso-Tapia[22], the ability to employ self-regulatory learning strategies is deemed vital to success in academic endeavors. The importance of self-regulated learning suggests a need to explore the possibility of equipping learners with self-regulatory capabilities to enhance their reading and eventually, their vocabulary learning. However, teachers lack effective ways to prepare learners to self-direct their own learning [23]. In classroom practice, some teachers may not recognize the need to motivate learners to increase their self-regulation in learning by giving students adequate guidelines for successfully executing complex assignments [24]. Under such a circumstance, students may not be able to self-assess their work and thereby unable to take metacognitive control of their academic work by monitoring their competence in learning to read or in learning new words [25]. In addition, some students may not be able to evaluate their learning, which may cause repeated experiences of failure [26]. In contrast, students who effectively monitor and evaluate the extent of their learning are more likely to secure a position allowing for intensifying or reorienting their studies to acquire a new skill through generating self-feedback [25, 27]. Hence, an important goal in classroom practice is to discern an effective method for helping students to achieve self-regulatory capabilities for their learning.

Previous studies using metacognitive guidance or group work have not addressed the possibility of incidental vocabulary learning occurring through reading. Despite studies on delineating the effects of metacognitive training on reading [17, 28], studies exploring the benefits of combining metacognitive guidance and group work in incidental vocabulary learning have yet to be conducted. The combination of these two kinds of instruction is assumed to result in internal information processing and elaboration processes which would eventually lead to an increased level of reading comprehension and incidental vocabulary learning. Metacognitive guidance is thus assumed to affect learners’ elaboration and learning processes while they work in groups. Additionally, group settings may afford learners more opportunities to engage in peer-to-peer interactions for reasoning and arguing about complex problems when comprehending texts and incidentally learning vocabulary, and subsequently self-regulate their learning after observing others’ behaviors [29]. However, this area of research has been overlooked in previous studies. In addition, the relation between form-meaning knowledge, e.g., form recall, meaning recall, form recognition, and meaning recognition, was not analyzed in this line of research. Administering multiple vocabulary assessments provides a more accurate picture of the range in vocabulary knowledge that can be gained incidentally through reading [2]. Hence, the present study is innovative in measuring reading and incidental vocabulary learning by involving EFL learners in either an individual or group setting reading task with or without the presence of self-regulatory prompts.

## Literature review

### Self-regulated learning and metacognition

Self-regulated learning (SRL) has been described as involving a process of self-generating thoughts, ideas, feelings, and actions for attaining one’s learning goals [30] or as the flexibly sequenced phases of recursive cognition of task perception, goal planning, enaction, and adaptation [31]. A controversy exists, however, on whether SRL should be described as the triadic analysis of self-regulation, representing the interactions of three forms of self-regulation: environment, behavior, and person [32] or as a multi-level model of self-regulation, focusing on the instruction of the self-regulatory processes [32]. Despite their differences, the common ground shared is that SRL involves more than detailed knowledge of a skill; however, SRL involves different processes, e.g., self-awareness, self-motivation, self-monitoring, and behavioral skill to realize goal setting and implement that knowledge appropriately [21, 33]. As argued by Tseng, Dörnyei, and Schmitt [34], the operational mechanism of self-regulatory capacity could be used for vocabulary learning.

Previous studies have proposed a connection between metacognition and self-regulated learning. According to Flavell [35], metacognition refers to an awareness of cognitive processes, related tasks and strategies, as well as an ability to regulate cognitive processes to plan, monitor, and assess one’s understanding and performance. Metacognition has been delineated as two basic components: Knowledge of metacognition and regulation of metacognition. The former involves the knowledge about a learner’s own cognition or cognition in general, including three types of knowledge: declarative knowledge (knowledge in understanding what factors may influence one’s academic success); procedural knowledge (knowledge in the use of different types of strategies for learning); and conditional knowledge (knowledge in using strategies for specific learning situations) [36]. The regulation of metacognition refers to learners’ ability to regulate their learning process, comprised of three basic regulatory skills: Planning, monitoring, and evaluating. Planning involves an ability to appropriately select strategies and effectively allocate resources for learning. Monitoring concerns an ability to observe task comprehension and performance targets. Evaluating regards an ability to appraise the regulatory process and the final product of a task [37]. Overall, learners with a wide range of metacognitive skills have been more self-regulated and thus likely to utilize and modify learning strategies and skills. In addition, the provision of metacognitive support might help learners self-regulate their learning and thus achieve better learning performance [38].

Metacognition and self-regulation seem to be synonyms. However, there are some subtle differences between the two constructs. While self-regulated learning explores how learners are able to evaluate themselves, identify their strengths and limitations, and then effectively employ appropriate strategies for learning goals, metacognition is more specific, involving learners’ active control over the cognitive processes while learning. Hence, metacognition is one aspect of self-regulated learning that also has a cognitive and motivational component. The commonality of these two constructs is the development of learners’ metacognitive skills through activities, including planning how to execute a learning task, monitoring comprehension, and evaluating the progress toward the completion of the learning task.

### The effects of metacognitive support on learning

Researchers have proposed the use of metacognitive support for helping learners overcome difficulties in self-regulating their learning. Nietfeld and Schraw [39] used prompts as a metacognitive strategy for helping to train students in understanding their own strengths and weaknesses in learning and discerning appropriate strategies for the assessment of their learning and the facilitation of their monitoring accuracy. In a similar vein, Davis [40] proposed that reflective-assessment prompts could help learners expand knowledge about their learning strengths and weaknesses, utilize appropriate strategies in assessing their learning, and draw casual inferences as to why progress was or was not being made. The findings from the studies reviewed above suggest that metacognitive prompts facilitate learners’ cognitive and affective evaluations of their current learning tasks and increase the chances for future engagement in learning. Thus, as argued by Bannert [41], learners that receive metacognitive prompts might retrieve a certain level of positive self-reactions to task performance. In contrast, learners without the support of metacognitive prompts might lose a sense of control over learning and their chances for engaging with future learning would decrease.

Nevertheless, claiming that metacognitive support always helps learners achieve a productive outcome in their learning would be misleading [42]. Some researchers have proposed the value of group work because social interactions among students positively impact learning [43]. Bandura [44] argued, for example, that group interactions might lead to more durable and transferable learning. For example, other-regulation, a part of social interactions, is essential to the development of self-regulation, i.e., the ability to plan, monitor, and control one’s behavior with the use of a set of rules that has been internalized. In other words, monitoring and controlling one’s own learning is related to an observation of how others monitor and control their behaviors. In a group setting, learners often ask and answer questions, model and contemplate strategies, offer and receive assistance and guidance from peers in the form of verbal and nonverbal signals which may help maintain the collaborative activity and improve, for example, reading skill [45]. Zhao, Li, Elliott, and Rueckl [46] also proposed the benefits of cooperative learning for reading aligning with Vygotsky’s [47] early proposal that interaction with more knowledgeable peers may help learners develop self-regulation.

Caveats for group work are also worth noting. For example, learners who are assigned to work in a group setting might lack positive interdependence, individual accountability, an equal opportunity to succeed, or interpersonal and social skills [48]. Thus, collaborative learning assignments have not always led to successful learning outcomes [49]. To overcome this limitation, researchers have pointed out that a metacognitive activity in a group setting might help increase confidence in building metacognitive skills [50, 51]. Cooperative-metacognitive learning refers to a learning setting in which learners offer their thoughts to peers for inspection, while also acting as a critic of their partner’s thinking and providing and receiving feedback as acts of facilitation, monitoring and regulation of their own and each other’s thinking and learning within a group setting. Kramarski and Mevarech [52] argued students working together in natural classroom settings allows for one’s thinking to be open to critique and refinement. Other studies have also shown the benefits of combining metacognitive prompts and group work to enhance learners’ metacognitive awareness and learning achievement. For example, in an experimental study, Teng [53] compared university-level learners who received or did not receive metacognitive training in either individual or group settings, finding learners who received metacognitive guidance in a group setting scored significantly higher on an English writing test than those who did not receive metacognitive prompts in a group setting, those who received prompts individually, and those who learned individually without metacognitive training.

Besides writing, previous studies have also been conducted in the fields of reading and vocabulary learning. For example, Shaaban [29] divided 44 English learners from Lebanon into two conditions: a condition focusing on group work and another focusing on individual work. The results did not show group work to be effective in improving vocabulary learning or reading comprehension. Shaaban [29] suggested a need to enhance the dynamics of interaction during cooperative learning, particularly for students who lack experience in cooperative learning and the social and collaborative skills necessary for cooperative learning. To address this need, Boulware-Gooden, Carreker, Thornhill, and Joshi [54] suggested adding metacognitive strategy training to improve vocabulary learning. Their results supported the effectiveness of metacognitive instruction in vocabulary learning success, e.g., generating synonyms, antonyms, and other related words. These results were also in line with Wilkinson’s [55] argument that working in groups helps enhance learners’ vocabulary learning.

### Research questions

The present study is innovative in that it aimed to explore university-level learners’ EFL reading comprehension and incidental learning of novel English words encountered during reading. Specifically, the first focus of the study was on comparing four treatment conditions—collaborative learning with prompts, collaborative learning without prompts, individual learning with prompts, and individual learning without prompts—on the performance of EFL reading and vocabulary learning. The second purpose of the study was to explore the size of the effect of the four treatment conditions on reading and the four dimensions of learning vocabulary (form recall, meaning recall, form recognition, meaning recognition). The present study was guided by two research questions:

Question 1: What are the effects of the four treatment conditions—formed by metacognitive prompts and learning setting (group vs. individual)—on EFL reading comprehension and incidental vocabulary learning?

Question 2: What is the size of the effect of the four treatment conditions on reading comprehension and the four dimensions of vocabulary learning?

## Method

The research reported in this manuscript was approved by the Nanning University Committee on the Use of Human & Animal Subjects in Teaching & Research. Written informed consent also was obtained from the participants. In 2014, the committee included 10 members that consisted of academic faculty staff (mainly professors) from different disciplines. All ethics forms related to research practices needed to be approved by this committee. This study was approved in 2014 and data collection was completed in 2014.

### Design

This study employed a 2×2 factorial design. The independent variables included the setting (individual versus group) and metacognitive prompts (learning with metacognitive prompts versus without metacognitive prompts). The dependent variables included a reading comprehension test and an English vocabulary test measuring the knowledge of the form-meaning relationship (see the Measurements section). The four treatment conditions are shown in Table 1.

**Table 1:** The factorial design employing four conditions.

Condition	With metacognitive prompts	Without metacognitive prompts
Group setting	Collaborative learning with prompts condition (CP) (*n* = 41)	Collaborative learning without prompts (CL) (*n* = 44)
Individual setting	Individual learning with prompts condition (IP) (*n* = 42)	Individual learning without prompts (IL) (*n* = 44)

Efforts were also made to rule out potential extraneous variables. This required the same: (a) exercises across the four treatment conditions; (b) experienced teacher, who had not taught the participants before, and was familiar with the procedure of this study, and took the role of instructor for the four treatment conditions; (c) instruction for the experiment; (d) material covered for instruction; (e) amount of time allocated for each condition; (f) assessment methods; (g) and classroom setting. The differences were in the respective interventional method and the materials that covered the unique components of each condition.

### Participants

This study focused on first-year students majoring in science and technology degrees as they are the largest number of EFL students at the university where the data was collected. Invitations were sent to 310 students in February 2014 and 220 students gave a positive response. The potential participants were administered a 100-point internal English test that measured reading, vocabulary, and writing. Among the 220 students, 30 students received scores above 80 points, 19 participants received a score below 65 points. The remaining 171 students received 70–75 points, indicating that they possessed an intermediate proficiency of English defined by the department of English at the university. Hence, the data from these 171 students (81 females and 90 males) were analyzed as they were the largest number of students that received test scores in the 70–75 point range at the research site and to some extent, represent first-year Chinese students with an intermediate English proficiency majoring in science and technology degrees. Participants ranged from 18–20 years of age. Their first language is Chinese. The descriptive statistics and ANOVA analysis of the internal English test scores helped ensure that the participants randomly placed in the four treatment conditions possessed a similar English language proficiency (*M* = 73.32, *SD* = 1.19, *p* = .68). The number of participants in each condition is presented in Table 1.

### Target items

A pool of 100 potential target English words were first selected from a textbook for the fourth-year students, these potential target words were not shown to the formal study participants to avoid the possible contamination of the research results due to familiarity with the tested target words. Instead, a pilot study was conducted with 50 students with similar English language proficiency and educational backgrounds aged between 18–20 years old. Among the 50 students, 26 were male and 24 were female. The pilot study participants were invited to review a checklist of the 100 potential words and tick unknown words on the list. The pilot study participants reported 63 words unknown to them. The researchers selected a total of 15 target words (see Table 2) from the 63 words based on the following criteria:

- The target words are all low frequency words, which were well beyond the participants’ language proficiency.
- The target words included an equal number of nouns, verbs, and adjectives, which are the most common parts of speech for words in natural English texts.
- The target words did not vary in word length (8–10 letters each).
- The target words had only one L1 translation (i.e., not polysemous).
- The target words could be easily and naturally be embedded into an English text containing high frequency words.
- The target words occurred only once in the English text, making word exposure frequency consistent in the four conditions.
- The target words did not occur in the exercises following the reading, as to avoid presenting more than one exposure of the target words to the learners.

**Table 2:** A list of target words.

Target words					
Noun:	Jubilation	Initiative	Pessimist	Eruption	Cowardice
Verb:	Intimidate	Stigmatize	Deprecate	Retaliate	Vandalize
Adjective:	Aggressive	Vulnerable	Draconian	Oblivious	Sumptuous

### Measurements

#### Reading comprehension test

Reading comprehension was measured through a 20-item reading test. This test aimed to measure the comprehension and recall of the main ideas from the text. The instructor and the first author assembled lists of main ideas from the text. Then during a discussion session, the first author and the instructor resolved discrepancies about the main ideas of the text through comparison and further discussion. The instructor then turned the main ideas into test items. Through consulting with experienced English teachers, question formats were developed to make tests consistent with the university textbook exercises. These included fill-in-the blank, true-or-false, and short answer questions (see S1 File). The three formats used in this test were selected as they follow the format of reading tests used in the Chinese EFL context. The Cronbach’s alpha for this test was .81, indicating good internal consistency. The participants had access to the reading material while completing the reading test.

#### Vocabulary test

One post-test assessing four dimensions of vocabulary learning adapted from a previous study [5] was administered to all participants. The Cronbach’s alpha for the four parts of test ranged from .71-.77, indicating an acceptable internal consistency. The test included:

Part I. Form recall

This part of the test measured learners’ ability to supply the written form of the L2 target word for a given meaning in the L1. Learners were required to supply the L2 target word to fit the L1 meaning prompt. The first letter of the L2 word was provided. For example:

I _______ (the target word is ‘intimidate’) 威脅

Part II. Meaning recall

This part of the test measured learners’ ability to supply the written L1 translation for the L2 target word. For example:

Intimidate________

Part III Form recognition

This was a meaning-matching test measuring whether learners could identify the matching L2 target word among four options for an L1 translation. For example:

威脅 A. Demotivate B. Intimidate C. Humiliate D. Destroy

Part IV Meaning recognition

This was a meaning-matching test measuring whether learners could identify the matching L1 translation among four options for an L2 target word. For example:

Intimidate A. 威脅 B. 冒險 C. 毀壞 D. 嫉妒

### Scoring system

Two experienced raters, neither teaching the participants, independently scored the responses for the tests. A third rater was invited in the event differences might arise between the two. In the reading comprehension test, the score was calculated based on the number of correctly answered items. One point was given for a correct answer while zero points were awarded for an incorrect answer. The maximum possible score for this test was 20 points.

The vocabulary test included four parts. In the first part, correct L1 target words provided by the participants were marked correct. Any misspelled word—for instance, ‘intimitate’ instead of ‘intimidate’—was marked incorrect. In the second part, semantically correct L2 meanings provided by the learners were marked correct. In the third and fourth part, answers for which learners correctly selected the correct option out of four were marked correct. In all cases in which no answer was provided or an incorrect answer was provided, the learners received zero points. In all cases in which the learners provided a correct answer, they were given one point. The maximum score for each dimension of the test was 15. No disagreements between the two raters emerged on marking the two tests.

### Procedure

This study was supported by the Department of English at one university in mainland China. At the beginning of this study, a consent form indicating participants’ responsibilities and benefits for taking part in the study was explained and then signed by the participants. It was explained to the participants that they would join a study using new learning and teaching methods and complete some reading exercises. However, they were not informed of the nature of their respective treatment conditions. Students participated in this research in exchange for extra course credit. None of the participants dropped out of the course or requested to terminate their participation in the study, although they were given the power to do so at any time without any consequences.

As suggested by the results of the pilot study, the time allowed for the experiment was 120 minutes. The participants first spent 30 minutes on silent reading of a 1500-word token text containing the 15 target words. This is a story reading text (“Uncle Theo”) taken from the textbook targeting fourth year students. As explained earlier, the text had been edited to embed the target words. This story text was chosen because it is assumed that participants, who were at the intermediate English proficiency level, may be more interested in reading a story than other genres. In addition, this is the type of textbook reading that is often required of EFL learners in China. After reading, the participants spent another 30 minutes reflecting on their understanding of the reading material. During this 30-minute reflection period, the reading material was still at their disposal. Participants in the first treatment condition were given metacognitive prompts in a group setting. Participants in the second treatment condition worked in a group setting without metacognitive prompts. Each group consisted of 4–6 students. The sub-group sizes were the result of students’ self selection. Participants in the third treatment condition were given metacognitive prompts and worked individually. Participants in the fourth treatment condition worked individually without metacognitive prompts. The metacognitive prompts included some self-addressed metacognitive questions, covering two components: knowledge of metacognition and regulation of metacognition (see Table 3). Prompts were printed on the participants’ worksheets and in the teacher’s guide. In the first treatment condition, metacognitive prompts were used by group members while reflecting on and discussing their reading comprehension, and by the teacher when providing help to the groups. In the third treatment condition, prompts were used individually when reflecting on the reading and by the teacher when providing help to individuals. Participants were informed that asking and answering the metacognitive prompts would help them to better understand what they read. The teacher’s help provided to the four conditions was consistent. The teacher only provided help when students initiated a request for help.

**Table 3:** Metacognitive prompts adapted from Teng [17].

Metacognition	Metacognitive self-addressed questions
Knowledge of metacognition	(i) What strength and weakness do I possess for this text reading? (ii)What factors have influenced our performance in comprehending this text? (iii)What strategies can be accessed efficiently for comprehending this text? (iv) How should we proceed to develop a solution for this reading exercise and in which way can we apply the strategies from previous learning experiences? (v) When and why should we use knowledge or strategies to improve our future reading of similar text?
Regulation of metacognition	(i) What are needed for planning, monitoring, and evaluating of this text reading?
	(ii) Do I set reasonable goals for this reading?
	(iii)What strategies should we use to plan the reading exercise?
	(iv) How should we organize our procedure for better reading comprehension? (v) How should we monitor this text-reading task? For example, what are the similarities and differences between the reading task at hand and tasks that we have solved in the past? (vi) How should we evaluate this reading task? Did I consider all the relevant information? What can I do to improve the reading proficiency?

Finally, the reading text was first collected and then participants were administered the unexpected reading and vocabulary tests. The participants were required to finish the tests within 60 minutes (the first 30 minutes was given for completion of the reading test and the second 30 minutes was given for completion of the vocabulary test). In terms of the vocabulary test, the participants first took the form recall test, then the meaning recall test, followed by the form recognition test, and finally the meaning recognition test. This process was aimed at minimizing any possible clues that the preceding test might provide for completing the subsequent test (i.e., practice effects). The teacher only distributed the next vocabulary test component to participants after having first collecting the previous component. Additionally, the order in which the target words were presented in each component of the vocabulary test was randomized and thus different. The participants had the reading materials at their disposal while completing the reading test but for the vocabulary test, the reading materials were collected. This was because we aimed for the reading test to measure comprehension and not content memorization. However, incidental vocabulary learning was evaluated as a “by product” of reading [56, 57, 58, 59]).

### Data analysis

The first research question’s aim was to investigate for probable differential effects of the four treatment conditions on the participants’ reading comprehension and incidental vocabulary learning. In terms of inferential statistical analysis for reading, a 2x2 two-way ANOVA was conducted to analyze participants’ reading comprehension outcomes for a statistically significant group difference and post hoc pairwise comparisons were then conducted to further tease apart the differences among the four participant groups.

Since the data for the four dimensions of knowledge of the form-meaning relationship largely conformed to the MANOVA requirement of correlations (from .35-.41), multicollinearity was avoided by applying a MANOVA to analyze the participants’ incidental vocabulary learning [60]. After MANOVA analysis, pairwise comparisons were used to compare incidental vocabulary learning outcomes by treatment conditions. In addition, univariate tests were used to determine whether there were any significant differences among the four dimensions of knowledge of the form-meaning relationship. Again, pairwise comparisons were performed to determine the relative difficulties of learning the four dimensions of knowledge of the form-meaning relationship.

The second research question’s aim was to examine the size of the effect of the four treatment conditions on reading and the four dimensions of knowledge of the form-meaning relationship incidentally learned through reading. Standard multiple regressions were conducted to explore the size of the effects of metacognitive prompts, setting, and their interaction on reading comprehension, as well as the learning of the four dimensions of knowledge of the form-meaning relationship.

In terms of the analyses, the familywise error rate, i.e., the probability of making a Type I Error, may increase due to repeated testing. We used a Bonferroni correction to control the possibility of a Type I error. The *p*-value were 0.05 for statistical significance. The effect sizes were calculated using partial η^2^.

## Results

### Effects of the four conditions on reading and incidental vocabulary learning

The results of the descriptive statistics of the reading comprehension test were first calculated. Results in Table 4 shows that participants in the Collaborative Learning with Prompts (CP) group achieved the highest reading comprehension scores (*M* = 15.31, *SD* = .907), followed by participants in the Collaborative Learning without Prompts group (CL) (*M* = 11.32, *SD* = .857), followed by the Individual Learning with Prompts (IP) group (*M* = 10.81, *SD* = .833), and lastly participants in the Individual Learning without Prompts (IL) group (*M* = 7.89, *SD* = .993).

**Table 4:** Descriptive statistics for the reading test (Maximum = 20).

Metacognitive prompts		*M*	*SD*	*N*
No	Individual learning	7.89	.993	44
	Collaborative learning	11.32	.857	44
	Total	9.60	1.957	88
Yes	Individual learning	10.81	.833	42
	Collaborative learning	15.32	.907	41
	Total	13.04	2.427	83
Total	Individual learning	9.31	1.730	86
	Collaborative learning	13.25	2.193	85
	Total	11.27	2.786	171

Next the 2x2 two-way ANOVA was conducted to examine whether there was a group effect on reading comprehension. Based on the Levene’s Test of Equality of Error Variance, the *p* value is larger than .05 (*F* = .326, *p* = .806). This shows that equal variances could be assumed and it was feasible to run a 2x2 two-way ANOVA. Results in Table 5 revealed a significant large effect of learning setting (individual vs. collaborative learning), *F*_(1, 170)_ = 830.542, *p* < .001, partial η^2^ = .83, as well as a significant large effect of metacognitive prompts, *F*_(1, 170)_ = 631.334, *p* < .001, partial η^2^ = .79. Results also revealed a significant medium interaction effect between metacognitive prompts and learning setting, *F*_(1, 170)_ = 15.247, *p* < .001, partial η^2^ = .08.

**Table 5:** Results of the 2x2 Two-way ANOVA for reading test.

Tests of Between-Subjects Effects						
Dependent Variable:	Reading					
Source	Type III Sum of Squares	*df*	Mean Square	*F*	Sig.	Partial η^2^
Metacognitive prompts	511.613	1	511.613	631.334	.000	.791
Learning setting	673.045	1	673.045	830.542	.000	.833
Metacognitive prompts × Learning setting	12.356	1	12.356	15.247	.000	.084
Error	135.332	167	.810			
Total	23035.000	171				
Corrected Total	1319.626	170				

Post hoc Bonferroni comparisons of the four conditions on reading comprehension test scores show that the Collaborative Learning with Prompts (CP) group significantly outperformed the Individual Learning without Prompts (IL) group (*p* < .001). In addition, Collaborative Learning with Prompts (CP) group significantly outperformed the Collaborative Learning without Prompts group (CL) (*p* < .001) and the Individual Learning with Prompts (IP) group (*p* < .001). However, there was no significant difference between the comprehension scores for the Collaborative Learning without Prompts (CL) group and Individual Learning with Prompts (IP) group (*p* = .058). This means that among the comprehension scores obtained by the four groups, the Collaborative Learning with Prompts (CP) group demonstrated the best reading comprehension performance (see Table 6).

**Table 6:** Post-hoc pairwise comparisons of the four conditions.

Group		Mean Difference	Standard Error	*p*	95% Confidence Interval for Difference	
					Lower Bound	Upper Bound
CL	IL	3.432*	.192	.000	2.919	3.944
	IP	.509	.194	.058	-.010	1.027
CP	CL	3.999*	.195	.000	3.477	4.521
	IL	7.431*	.195	.000	6.909	7.952
	IP	4.508*	.198	.000	3.980	5.035
IP	IL	2.923*	.194	.000	2.405	3.442

*Note*. CL = Collaborative learning without prompts

CP = Collaborative learning with prompts

IL = Individual learning without prompts

IP = Individual learning with prompts

* *p* < .001

The descriptive statistics for the vocabulary tests were also calculated. As shown in Table 7, the average total score for each of the four treatment conditions ranged from 2.77−8.94 out of a maximum 15. The participants in the Collaborative Learning with Prompts (CP) group achieved the highest incidental vocabulary learning outcomes. In contrast, the participants in the Individual Learning without Prompts (IL) group achieved the lowest incidental vocabulary learning outcomes. In relation to the four dimensions of knowledge of the form-meaning relationship, the total scores ranged from 2.37 to 9.6 out of a possible 15. Meaning recognition appeared to be the easiest dimension for all participants to learn incidentally. In contrast, the knowledge of form recall was the most challenging dimension for all participants.

**Table 7:** Word learning scores by the four dimensions of knowledge of the form-meaning relationship (Maximum = 15).

Dependent variable	Collaborative learning with prompts (CP) (*n* = 41)	Collaborative learning without prompts (CL) (*n* = 44)	Individual learning with prompts (IP) (*n* = 42)	Individual learning without prompts (IL) (*n* = 44)	Total (*n* = 171)
Form recall	4.65 (.85)	2.27 (.62)	2.04 (.73)	.65 (.56)	2.37 (1.59)
Meaning recall	7.21 (.65)	4.13 (.63)	4.14 (.64)	2.20 (.63)	4.38 (1.89)
Form recognition	10.31 (.87)	7.11 (.81)	7.07 (.77)	3.15 (.74)	6.85 (2.66)
Meaning recognition	13.58 (.86)	10.27 (.84)	9.76 (.87)	5.09 (.74)	9.6 (3.12)
Total score	8.94 (3.45)	5.94 (3.13)	5.75 (3.02)	2.77 (1.74)	

*Note*. SD are in parentheses.

The next step was to run a MANOVA for the vocabulary learning outcomes. Box’s Test of Equality of Covariance Matrices showed that the variance-covariance matrices were not equal. Following this violation of the null hypothesis that the observed covariance matrices of the dependent variables were equal across groups, we used Pillai’s criterion for multivariate tests. Results are presented in Table 8. Results showed a significant large effect of metacognitive prompts on vocabulary learning, *F*_(1, 169)_ = 326.396, *p* < .001, partial η^2^ = .88, a significant large effect of learning setting on vocabulary learning, *F*_(1, 169)_ = 369.472, *p* < .001, partial η^2^ = .90, and a significant large effect of the interaction between metacognitive prompts and learning setting on incidental vocabulary learning, *F*_(1, 169)_ = 45.124, *p* < .001, partial η^2^ = .52.

**Table 8:** MANOVA results for incidental vocabulary learning outcomes.

Multivariate Tests^a^							
Effect		Value	*F*	Hypothesis *df*	Error *df*	*p*	Partial η^2^
Metacognitive prompts	Pillai’s Trace	.888	326.396^b^	4.000	164.000	.000	.888
Learning setting	Pillai’s Trace	.900	369.472^b^	4.000	164.000	.000	.900
Metacognitive prompts × Learning setting	Pillai’s Trace	.524	45.124^b^	4.000	164.000	.000	.524

^a^Design: metacognitive prompts + learning setting + metacognitive prompts x learning setting

^b^Exact statistic

Following the multivariate tests, univariate tests were conducted to explore the effects of the four conditions on the incidental learning of the four dimensions of knowledge of the form-meaning relationship. Results of the univariate tests presented in Table 9 revealed a significant effect of metacognitive prompts, a significant effect of learning setting, and a significant interaction effect between metacognitive prompts and learning setting; these results were consistent for the incidental learning of the four dimensions of form-meaning knowledge. Of particular interest is the significant large effect of metacognitive prompts on form recall [*F*(1, 169) = 310.775, *p* < .001, partial η^2^ = .65], meaning recall [*F*(1, 169) = 836.146, *p* < .001, partial η^2^ = .83], form recognition [*F*(1, 169) = 656.306, *p* < .001, partial η^2^ = .79], and meaning recognition [*F*(1, 169) = 980.346 *p* < .001, partial η^2^ = .85]. There was also a significant large effect of learning conditions on form recall [*F*(1, 169) = 389.338, *p* < .001, partial η^2^ = .70], meaning recall [*F*(1, 169) = 856.102, *p* < .001, partial η^2^ = .83], form recognition [*F*(1, 169) = 652.916, *p* < .001, partial η^2^ = .79], and meaning recognition [*F*(1, 169) = 1247.306, *p* < .001, partial η^2^ = .88]. Lastly, there was a significant medium interaction effect of metacognitive prompts and learning conditions on form recall [*F*(1, 169) = 21.697, *p* < .001, partial η^2^ = .11], small effect on meaning recall [*F*(1, 169) = 8.299, *p* < .001, partial η^2^ = .04], large effect on form recognition [*F*(1, 169) = 34.114, *p* < .001, partial η^2^ = .17], and large effect on meaning recognition [*F*(1, 169) = 28.380, *p* < .001, partial η^2^ = .14].

**Table 9:** Results on the univariate tests following multivariate tests.

Source		Type III Sum of Squares	*df*	Mean Square	*F*	*p*	Partial η^2^
Metacognitive prompts	Form recall	152.109	1	152.109	310.775	.000	.650
	Form recognition	269.235	1	269.235	656.306	.000	.797
	Meaning recall	540.650	1	540.650	836.146	.000	.834
	Meaning recognition	680.571	1	680.571	980.346	.000	.854
Learning setting	Form recall	190.561	1	190.561	389.338	.000	.700
	Form recognition	267.845	1	267.845	652.916	.000	.796
	Meaning recall	553.554	1	553.554	856.102	.000	.837
	Meaning recognition	865.898	1	865.898	1247.305	.000	.882
Metacognitive prompts x Learning setting	Form recall	10.620	1	10.620	21.697	.000	.115
	Form recognition	13.995	1	13.995	34.114	.000	.170
	Meaning recall	5.366	1	5.366	8.299	.004	.047
	Meaning recognition	19.702	1	19.702	28.380	.000	.145

The post-hoc analyses of the adjusted mean scores based on the pairwise comparisons shown in Table 10 indicated that, in terms of form recall vocabulary knowledge, Collaborative Learning with Prompts (CP) yielded significantly higher scores compared to Collaborative Learning without Prompts (CL) (*p* < .001), Individual Learning with Prompts (IP) (*p* < .001), as well as Individual Learning without Prompts (IL) (*p* < .001). Likewise, the acquisition of meaning recall vocabulary knowledge was significantly higher for the Collaborative Learning with Prompts (CP) group compared to the Collaborative Learning without Prompts (CL) group (*p* < .001), the Individual Learning with Prompts (IP) group (*p* < .001), as well as the Individual Learning without Prompts (IL) group (*p* < .001). The same pattern of results was shown for the acquisition of form recognition vocabulary knowledge, with significantly higher scores for the Collaborative Learning with Prompts (CP) group compared to the Collaborative Learning without Prompts (CL) group (*p* < .001), the Individual Learning with Prompts group (IP) (*p* < .001), as well as the Individual Learning without Prompts (IL) group (*p* < .001). The same pattern in the results was shown for the acquisition of meaning recognition vocabulary knowledge, for which the Collaborative Learning with Prompts (CP) group yielded significantly higher scores than the Collaborative Learning without Prompts (CL) group (*p* < .001), the Individual Learning with Prompts (IP) group (*p* < .001), as well as the Individual Learning without Prompts (IL) group (*p* < .001). Taken together, these results indicate that the provision of metacognitive prompts in a group setting was an effective means for incidental vocabulary learning. In addition, participants using metacognitive prompts in an individual setting also consistently exhibited significantly higher scores for incidental learning than the participants without prompts in an individual setting. This result was shown irrespective of the dimension of vocabulary knowledge being assessed. However, significant differences between Collaborative Learning without Prompts (CL) and Individual Learning with Prompts (IP) groups were not detected in the four conditions (*p*>.05).

**Table 10:** Post-hoc analyses of the four conditions on the four dimensions of incidental vocabulary learning.

Pairwise Comparisons							
Dependent Variable			Mean Difference	Standard Error	*p*	95% Confidence Interval for Difference	
						Lower Bound	Upper Bound
Form recall	CP	CL	2.386*	.152	.000	1.980	2.791
		IL	3.999*	.152	.000	3.594	4.405
		IP	2.611*	.154	.000	2.201	3.021
	IL	CL	-1.614*	.149	.000	-2.012	-1.215
		IP	-1.389*	.151	.000	-1.791	-.986
	IP	CL	-.225	.151	.826	-.628	.178
Form recognition	CP	CL	3.083*	.139	.000	2.712	3.454
		IL	5.015*	.139	.000	4.644	5.386
		IP	3.077*	.141	.000	2.701	3.452
	IL	CL	-1.932*	.137	.000	-2.296	-1.567
		IP	-1.938*	.138	.000	-2.307	-1.569
	IP	CL	.006	.138	1.000	-.362	.375
Meaning recall	CP	CL	3.203*	.175	.000	2.737	3.669
		IL	7.158*	.175	.000	6.692	7.624
		IP	3.246*	.177	.000	2.774	3.717
	IL	CL	-3.955*	.171	.000	-4.412	-3.497
		IP	-3.912*	.173	.000	-4.376	-3.449
	IP	CL	-.042	.173	1.000	-.505	.421
Meaning recognition	CP	CL	3.313*	.181	.000	2.830	3.796
		IL	8.494*	.181	.000	8.012	8.977
		IP	3.823*	.183	.000	3.335	4.312
	IL	CL	-5.182*	.178	.000	-5.656	-4.708
		IP	-4.671*	.180	.000	-5.151	-4.191
	IP	CL	-.511*	.180	.030	-.991	-.031

*Note*. CL = Collaborative learning without prompts

CP = Collaborative learning with prompts

IL = Individual learning without prompts

IP = Individual learning with prompts

* *p* < .001

Finally, a repeated measures ANOVA was run revealing that the differences between scores of the four dimensions of knowledge of the form-meaning relationship was significant, *F* (3, 167) = 11.72, *p* < .001, partial η^2^ = .68. Pairwise comparisons revealed that learners achieved significantly higher scores for meaning recognition vocabulary knowledge than form recognition vocabulary knowledge (*p* < .001), meaning recall vocabulary knowledge (*p* < .001), and form recall vocabulary knowledge (*p* < .001). This result indicated that, irrespective of treatment condition, form recall of unknown words was the most challenging dimension for the participants to learn incidentally while the meaning recognition of unknown words was the least challenging.

### The size of effect of the four conditions on different dimensions of the knowledge of the form-meaning relationship

To answer the second research question, standard multiple regression analyses were conducted with group/individual setting, with/without metacognitive prompts, and the interaction of two as independent variables. The deviations from zero for each variable was not equal possibly due to an unequal number of participants in the treatment conditions. In addition, the distribution of form and meaning recall test results violated assumptions of normality for skewness. Transformations of the two variables, which were conducted by calculating the square root of each, resulted in normal distributions. The results of the standard multiple regression analyses for the four dimensions of the knowledge of the form-meaning relationship are presented in Table 11.

**Table 11:** Standard multiple regression of prompts, settings, and prompts x setting for reading comprehension and the four dimensions of knowledge of the form-meaning relationship.

Dependent variables	Independent Variables	*B*	Std. error	*β*	sr^2^
Reading comprehension	Prompts	2.923**	.194	.526	.576
	Setting	3.432**	.192	.618	.657
	Prompts×setting	1.076**	.275	.165	.084
	Intercept = 7.886				
	R^2^ = .897				
	Adjusted R^2^ = .896				
	R = .947				
Form recall	Prompts	1.389*	.151	.438	.336
	Setting	1.614*	.149	.509	.412
	Prompts×setting	.997	.214	.268	.114
	Intercept = .659				
	R^2^ = .810				
	Adjusted R^2^ = .807				
	R = .900				
Meaning recall	Prompts	3.912**	.173	.736	.752
	Setting	3.955**	.171	.745	.761
	Prompts×setting	-.709*	.246	-.114	.047
	Intercept = 3.159				
	R^2^ = .910				
	Adjusted R^2^ = .909				
	R = .954				
Form recognition	Prompts	1.938**	.138	.512	.540
	Setting	1.932**	.137	.510	.545
	Prompts×setting	1.145**	.196	.258	.169
	Intercept = 2.206				
	R^2^ = .888				
	Adjusted R^2^ = .886				
	R = .942				
Meaning recognition	Prompts	4.671**	.180	.744	.801
	Setting	5.182**	.178	.826	.835
	Prompts×setting	-1.358**	.255	.185	.145
	Intercept = 5.091				
	R^2^ = .61				
	Adjusted R^2^ = .58				
	R = .77**				

Note.

*p < .05

**p < .001

As shown in Table 11, there were significant main effects for metacognitive prompts and setting and a significant Prompts x Setting interaction for reading comprehension. The adjusted R^2^ indicated that 89% of the variability could be predicted by the three independent variables. More specifically, metacognitive prompts accounted for 57% of the variability, learning setting accounted for 65% of the variability, and the interaction between prompts and settings accounted for an additional 8.4% of the variability. The introduction of metacognitive prompts and collaborative learning increased reading comprehension. The main effects of collaborative learning over individual learning and the presence metacognitive prompts over the absence of metacognitive prompts on reading comprehension were clearly shown. In addition, the interaction between metacognitive prompts and learning setting on reading comprehension was also evident.

In terms of the four dimensions of knowledge of the form-meaning relationship, the adjusted R^2^ for the form recall test indicated that 81% of the variability was predicted by the three independent variables. In detail, prompts accounted for 33% of the variability, setting accounted for 41% of the variability, and the interaction between prompts and settings accounted for an additional 11%. A slight difference was shown for the meaning recall test. The adjusted R^2^ indicated that 90% of the variability was predicted by the three independent variables. In detail, prompts accounted for 75% of the variability, setting accounted for 76% of the variability, and the interaction between prompts and settings accounted for an additional 4.7%. In terms of the form recognition test, the adjusted R^2^ indicated that 88% of the variability was predicted by the three independent variables. In detail, prompts accounted for 54% of the variability, setting accounted for 54% of the variability, and the interaction between prompts and settings accounted for an additional 16%. In terms of the meaning recognition test, the adjusted R^2^ revealed that 58% of the variability was predicted by the three variables. In detail, prompts accounted for 80% of the variability, setting accounted for 83% of the variability, and the interaction between prompts and settings accounted for an additional 14%. Overall, the presence of metacognitive prompts increased incidental vocabulary learning, but more so for meaning recall, form recognition, and meaning recognition (*p* < .001). Also, collaborative learning increased incidental vocabulary learning more than individual learning, but more so for meaning recall, form recognition, and meaning recognition. The main effects of collaborative learning over individual settings and the presence of metacognitive prompts over the absence metacognitive prompts were clearly evident. In addition, the interaction between metacognitive prompts and setting on form recognition and meaning recognition could be explained by this significant difference (*p* < .001). The interaction between metacognitive prompts and setting on meaning recall could also be explained by this significant difference (*p* < .05); however, the interaction between metacognitive prompts and setting on form recall vocabulary knowledge was not shown to be significant (*p*>.05)

## Discussion

This study addressed whether the provision of metacognitive prompts in group settings would improve learners’ reading comprehension and incidental learning of novel words. Findings supported that using metacognitive prompts in a group setting significantly improved learners’ reading comprehension. The significant interaction between prompts and settings indicated that using metacognitive prompts in a group setting does have an additive impact on enhancing the incidental learning knowledge of the form-meaning relationship to a greater extent than the other treatment conditions investigated.

Several issues related to the comparisons between the treatment conditions must be considered. First, the participants who received metacognitive prompts outperformed those without prompts for reading comprehension and incidental vocabulary learning. This outcome may be because metacognitive prompts provided a catalyst for learners to foster higher-order thinking skills and elaborate more on information during the execution of self-regulatory learning strategies. The facilitation of strategies could have possibly enhanced participants’ reflection skills that aided in their identifying contextual clues for comprehension, integrating multiple perspectives in monitoring and regulating their own reading processes, and boosting their potential vocabulary learning outcomes [61]. Unsuccessful readers, however, lack such skills, which negatively affects textual level comprehension [28, 62]. Having said this, we see the value of metacognitive prompts used by the participants in the current study with specific reference to the context in which reading takes place. The metacognitive prompts seemed to help participants focus more on text meaning, and therefore, they discerned the metacognitive processes involved with reading and figured out when and how they need to ‘predict’, ‘summarize’, ‘infer’, and ‘monitor’ their comprehension processes. The metacognitive prompts may have provided a springboard for the participants to infer text meaning via contextual and linguistic clues, and this may be one of the reasons the participants outperformed those participants without metacognitive prompts. Those participants without metacognitive prompts may have remained at the perceptual processing stage [63]. Or else, they might have just made wild speculations and guesses about what they read [62].

Second, results indicated that when participants worked together in group settings to accomplish shared learning goals, higher individual achievement was shown. Thus, collaborative efforts lead to a better learning outcome and ensures cognitive development [50, 53, 64]. It seems that a collaborative learning setting may enable participants to engage in peer interactions and motivate them to argue, reason, and negotiate while reading [45, 65]. It seems that collaborative learning may build and enhance learners’ capacity to pool ideas and facilitate each other with feedback and identify and solve problems that arise while reading [46, 65]. Therefore, a collaborative learning setting appears to be beneficial to learners’ reading because learners may be more engaged with the reading.

Finally, the participants receiving metacognitive prompts in a group setting outperformed the participants in the other three treatment conditions. The improved performance included reading comprehension and incidental learning of form recall, meaning recall, form recognition, and meaning recognition of vocabulary knowledge. An interpretation of this outcome is that the types of metacognitive prompts were appropriate for a collaborative learning setting. Specifically, the metacognitive prompts acquainted learners with the necessary actions to search for various information, monitor and evaluate this process, conduct argumentation, reason, and problem-solve, which enabled participants to engage in peer interaction and further motivated them to debate and reason with one another to understand the text while simultaneously grasping the gist of some difficult words [18, 19, 33, 54, 65]. These effects suggest that success in a group learning setting may be rooted in metacognitive processes. Therefore, the learners who were provided metacognitive prompts in a group setting outperformed even those learners who also learned in a group setting but without metacognitive prompts.

Findings from this study regarding the benefits of providing metacognitive prompts in a group setting for promoting reading and incidental vocabulary learning are also supported by previous research. In a group setting, Dahl [66]) and Shih and Reynolds [65] observed students reflecting, verbalizing to others about their learning process, and synthesizing the new learning that allowed them to produce a dramatic skit to share with an entire class. The collaborative interactions within a group dynamic acted as a stimulus for learners to execute metacognitive strategies associated with their cooperative tasks and stimulate a problem-solving process for observing discrepancies between actual and intended text comprehension. A similar process might have helped the participants in the current study to detect the problems arising while reading and then correct the problems, which has been shown as an effective means for enhancing text comprehension [54] as well as the understanding of unknown vocabulary [55]. The results from the current study support the social interdependence theory that underscores social support and classroom interaction for the enhancement of the psychosocial adjustments of learners [67; 68]. However, the results in the present study were not in line with Shaaban’s [29] study, which indicated that collaborative learning did not always lead to expected reading comprehension and vocabulary learning results. In his study, participants in a group setting received reading texts on expert topics along with accompanying worksheets that were to be used to assist with comprehension of concepts and ideas presented in the reading materials. In contrast, the learners in the present study received metacognitive prompts in a cooperative learning setting to facilitate their reading comprehension process. The metacognitive prompts provided to participants in the current study may have provided the benefits of improved judgment in discerning of the subject matter and initiating a process of inquiry [39]. Hence, the provision of metacognitive prompts in a group setting may initiate academic support from peers as they negotiated meaning, providing members of the group a rich resource for solving problems inherent in comprehending texts, thereby guiding them to achieve common goals by reflecting on how to recognize and recall a certain level of vocabulary knowledge. As asserted by Davis [40], learners who are engaged in reflection are better able to expand their repertoire of knowledge or ideas, determine weaknesses in their current knowledge, and link what they already know to exploring what they do not know.

Participants in the current study who received metacognitive prompts in a group setting may have also been placed in a position to receive feedback from external resources that encouraged engagement in the group’s activities, allowing them access to evaluate their level of knowledge, and be more committed to reaching their goals [53]. As proposed in previous research [49], merely putting learners into small groups does not automatically lead to interactive group work and effective learning. Some learners may lack positive interdependence and individual accountability [69], or an inability to plan, monitor and reflect upon their learning processes [48], which may further affect cooperative learning. Evidenced by the findings in the present study, we argue that the deficit of having collaborative learning may be compensated by the provision of metacognitive prompts. These prompts directed the participants in the current study to apply metacognitive strategies in fostering explanations and engaging in discussions, reflect upon the task at hand, and direct peer instruction to improve self-regulated learning [41]. This progression also led to deeper processing of information, facilitation of higher-order thinking skills, and ultimate improvement of intended learning results.

The results of the present study also highlight the value of self-regulatory learning for reading comprehension and learning new words incidentally encountered during reading. The prompts in the current study were a series of questions used to provide feedback for the enhancement of learners’ self-regulated capacity. The participants who received the prompts were encouraged to respond to questions that asked how well they could understand the text, how confident they were about their reading comprehension, and how they would assess their reading strengths and weaknesses. The prompts helped the participants to self-regulate their learning, which can be explained by Zimmerman and Moylan’s [30] self-regulatory feedback model. The metacognitive prompts, which focused on planning, monitoring, and evaluating, seemed to assist the participants in the following ways. First, the prompts helped the participants to figure out how to approach the reading task during a planning phase. With the help of the prompts, the participants analyzed the characteristics of the reading task, assessed their capacity to perform it, and established goals and a plan on how to complete the task [16]. Second, the prompts helped the participants self-observe and self-control during the performance phase [34]. This likely maintained participants’ concentration and helped them keep track of their reading progress. Third, the prompts provided guidelines for the participants to self-reflect on their learning. During this self-reflection phase, the participants began building a capacity to judge their learning and formulate reasons for their ability to comprehend the reading text. The participants may have reacted cognitively to their own attributions by judging their success and failure as opportunities to improve reading comprehension. In the present study, the frequency of exposure to the new words was controlled to one occurrence. Since we know from previous research that one exposure is generally regarded as “insufficient” for incidental word learning in most cases [7, 9, 10, 12, 15, 69, 70]), but participants in the current study might have discussed the target words thereby increasing exposure. This lends credibility to the argument that metacognitive prompts training could be effective in enhancing incidental vocabulary learning.

However, Panadero and Alonso-Tapia [22] argued that the self-regulatory feedback model by Zimmerman and Moylan [30] did not cover the social aspects of regulation of learning. The results from the present study seemed to support the role of group work in enhancing self-regulated learning. The participants with prompts in a group setting appeared to have self-regulated their learning through scaffolding provided by their peers. This suggests that self-regulation happened during the collaborative interactions among peers [71]. Hence, the self-regulatory feedback model should not only focus on how learners self-regulate their learning but also how they explore the synergies and interactions belonging to the regulation while completing group work. This determination suggests a need to explore the three types of regulation [71]): (a) self-regulated learning, with a focus on how individuals adapt to it for the realizations of their goals; b) co-regulated learning, with an emphasis on the interaction between two or more individuals in a group; and (c) socially shared regulated learning, in which a joint management of the group members is employed to achieve negotiated and shared goals. Although the use of metacognitive prompts in addressing the three types of regulation was not covered in the present study, we hope other researchers will consider investigating additional types of metacognitive prompts in future studies.

The results from the present study highlight that learners gain different levels of mastery over lexical items. Although the four dimensions of knowledge of the form-meaning relationship were interrelated and holistically connected, the process of learning vocabulary was observed to follow an incremental nature[8]. In addition, echoing Teng [10], complete mastery of a lexical item was found to involve establishing a map that linked form and meaning, but not all of which can be learned completely from completion of a single reading activity because each word can vary in the degree to which receptive/productive knowledge has been mastered. The present study results suggest that some dimensions of knowledge of the form-meaning relationship were mastered before others regardless of the treatment condition. For example, just from exposure to a new word in a written text, word meaning recognition was more likely to be picked up than form recognition, meaning recall, and form recall. However, findings in the present study could not confidently explain how the different types of knowledge of the form-meaning relationship developed in relation to each other. Perhaps it was because the measures employed in the present study were limited as they could only examine the learning outcome of multiple types of vocabulary knowledge concurrently. Still, there was a pattern in the results that indicated the combination of a group setting along with the metacognitive prompts led to higher rates of incidental vocabulary learning—this pattern in the data was present regardless of the type of vocabulary knowledge assessed. More sensitive studies are needed for exploring the zero→partial→precise development of vocabulary knowledge.

## Conclusion, limitations, and implications

In summary, the findings in the present study established that learners working in groups and with metacognitive prompts were more likely to improve reading comprehension and to incidentally learn more unknown words from reading. When considering the interactive effects of prompts and group settings, it was revealed that the prompts may have directed the learners to conduct self-reflection and take metacognitive control of their knowledge, which likely illuminated their sense of strengths and weaknesses. In addition, group work appeared to help learners internalize and activate metacognitive processes for reading and vocabulary learning.

The present study provides implications for classroom practice using metacognitive prompts. First, learners may benefit from either metacognitive prompts or collaborative group learning as a part of their studies. Incorporating metacognitive prompts or arranging participants to study in a group may be a palatable means for learners to achieve an extended level of performance in reading comprehension and new word learning. Second, self-regulated learning relates to how teachers gradually transfer responsibility to students. The learners that participated in the current study were required to assume responsibility for the development of their reading and vocabulary learning skills. Finally, as other researchers [23; 28; 65] have cautioned, such an approach may require time on part of the teachers to establish effective means of instruction through metacognitive prompts to enhance learners’ reading comprehension. The results of the current study revealed that this endeavor by the classroom teacher was worthwhile as the engagement of metacognitive prompts encouraged the learners to reflect on their reading process and solve their problems resulting from an inadequacy of language knowledge to comprehend and understanding textual information. Results further revealed that the employment of comprehension reading strategies (e.g., planning, monitoring, and evaluating strategies) in a collaborative learning setting encouraged the learners to share, facilitate, integrate, synthesize, and control their own reading processes. Therefore, it is of value for reading teachers to familiarize their learners with knowledge and strategies of metacognition to help improve learners reading comprehension [72]. Teachers might also need to guide and scaffold learners to use those strategies in a collaborative learning setting to ensure that peer feedback facilitates higher levels of reading comprehension; this is especially important for learners with limited linguistic knowledge [23].

Limitations also exist in this study. First, the participants were not exposed to reading tasks of varying complexity, which would have allowed for further exploration of potential interactive effects of metacognitive prompts and additional group dynamics. However, this may be better accomplished in a separate study as adding reading text complexity as an independent variable would have also increased the complexity of the research design. Second, this study was limited to 171 tertiary level students at one university in China. Differences in performance between genders were not analyzed and the generalizability of the findings may be limited to Chinese learners of English. Third, group membership was determined through random assignment by the classroom teacher. Students who experienced any personality conflict while working with each other might be reluctant to continue group work or at the very least have their participation affected to some degree. Freedom to form a group of familiar students in one’s classroom might produce different results. Future studies should also analyze team heterogeneity, i.e., students who differ in their ability to work together as a group, which might further encourage and advance learning. Fourth, this study aimed to measure four dimensions of the knowledge of form-meaning relationships. However, test effects may have occurred and influenced the findings. In addition, this study lacked a pretest and a delayed post-test. As it is within the incidental vocabulary learning research methodology paradigm to not administer pre-tests if proper pilot testing has been conducted [2], we assumed that a pretest was not necessary. However, including a delayed post-test could have helped us to understand more about the participants’ ability in transferring what they had learned to new tasks or to determine whether the presence or absence of metacognitive prompts could help to resist decay in vocabulary knowledge [73]. We also admit that it would have been difficult to follow the participants recruited in the current study for a delayed posttest as they would not have been enrolled in the English courses at the appropriate time that posttests should have been administered. It is pertinent that future studies tackle this issue. Fifth, the present study lacked a qualitative approach to observe group interaction, for example through language-related episodes, which might help in pinpointing the kinds of responses or actions that are linked to better achievement in incidental vocabulary learning [72]. The findings merit future replication research that can build upon the findings of the current study by recruiting English learners with varying levels of language proficiency that could be observed over a longer period of time under a number of potential treatment conditions. Lastly, although it was ensured that the text given to participants only contained words from the most frequent 2,000 words of English, there is still the possibility that there could have been some words other than the target words that could have been unknown by some of the participants. Despite these limitations, the present study does break new ground in that the results showed a clear indication that collaborative-metacognitive training administered by a teacher can help enhance learners’ reading comprehension and incidental vocabulary learning through reading.

## Supporting information

S1 File This is the reading text, assessments, and assessments. (DOCX)

S2 File This is the data. (SAV)
