# Supplementary material for: Effects of individual and group metacognitive prompts on EFL reading comprehension and incidental vocabulary learning
Source: PLoS One. 2019 May 22;14(5):e0215902. doi: 10.1371/journal.pone.0215902 (PMC6530832; doi:10.1371/journal.pone.0215902)
Supplement: S1 File — (DOCX) [file pone.0215902.s001.docx]

Appendix

Directions: Please read this text within 30 minutes. After that, please finish the exercises related to this text.

**My Uncle Theo**

My Uncle Theo is a man with real brains. He is my oldest uncle, a tall, thin, grey-haired man whose thoughts are always on learning and nothing else. He is quiet, gentle and absent-minded and with about as much sense as a child where money is concerned. He is positive, and always declares that only a foolhardy pessimist would deny the wonderful opportunities that lie ahead. He has a reputation for being kind and not intimidating those innocent people. He believes that everyone needs to focus on trying to make sure that people don’t stigmatize the community even further. Well, he applied for a post in Camford University. It was a very good post and there were hundreds of candidates who applied for it, and about fifteen, including Theo, were asked to go to be interviewed. But he said applying for a job needs a good mood. Anybody who could not face up to failure in a job is cowardice rather than compassion.

Camford is a very small town; there is only one hotel in it, and this was so full that they had to put many of the candidates two in a room. Theo was one of these, and the man who shared the room with him was a self-confident fellow called Adams, about twenty years younger than Theo, with a loud voice, and a laugh that you could hear all over the hotel. But he was a clever fellow all the same and had a good post in Iscariot College, Narkover. Well, the Dean, that’s the head of the department of the university, and the committee interviewed all the candidates, and, as a result of the interview, the number was reduced to two, uncle Theo and Adams. The committee couldn’t decide which of the two to take, so they decided to make their final choice after each candidate had given a public lecture in the college lecture-hall. The subject Adams had to speak on was “The Civilization and modern society”. The subject for uncle Theo was “Nature”. The lecture had to be given in three days’ time.

Well, for three days Uncle Theo never left his room. He worked day and night at that lecture, writing it out and memorizing it, almost without eating or sleeping. Adams didn’t seem to do any preparation at all. You could hear his voice and his laughter in the bar where he had a crowd of people round him. He came to his room late at night, asked Uncle Theo how he was getting on with his lecture, and then told him how had spent the evening playing bridge, or at the music-hall. He ate like a horse and slept like a log; and Uncle Theo sat up working at his lecture.

The day of the lecture arrived. They all went into the lecture-room and Theo and Adams took their seats on the platform. And then, Theo discovered, to his horror, that the typewritten copy of his speech had disappeared. The dean said they call on the candidates in alphabetical order, Adams first; and, with despair in his heart, Theo watched Adams calmly take the stolen speech out of his pocket and read it to the professors who were gathered to hear it. Adams mentioned a lot about modern society and civilization. He said, “Last year, there were big protests over draconian new laws to regulate violence. We have many times more police wandering around making sure aggressive people don’t vandalize our community. In the wider sense, we deprecate any forms of bigotry and sectarianism, we take a very strong view that these types of activity are unacceptable in a modern society. It could do even more damage to the modern society if—as is all too likely—people take actions to retaliate. We need compassion.” And how well he read it! Even Theo had to admit that he couldn’t have read it nearly so eloquently himself, and Adams finished there was a great burst of applause. Adams bowed and smiled, and sat down.

Now it was Theo’s turn. But what could he do? He had put everything he knew into that lecture. His mind was too much upset to put the same thoughts in another way. With a burning face he could only repeat, word for word, in a low, dull voice the lecture that Adams had spoken so eloquently. He talked about the nature and the eruption and suggested the importance of monitoring apparatus in recording an earthquake and formation of magma. Uncle Theo also mentioned how vulnerable people are in front of natural catastrophe. There was hardly any applause when he sat down.

The Dean and the committee went out to decide who the successful candidate was, but everyone was sure what their decision would be. Adams leaned across to Theo and patted him on the back and said, smilingly, “Hard luck, old fellow, but, after all, one of us could win.”

Then the Dean and committee came back. “Gentlemen,” then Dean said, “the candidate we have chosen is Mr. Hobdell.” Uncle Theo had won! The audience was completely taken by surprise, and the Dean continued, “I think I ought to tell you how we arrived at that decision. We were all filled with admiration at the learning and eloquence of Mr. Adams. I was greatly impressed; I didn’t think he had it in him. But, you will remember, Mr. Adams read his lecture to us. When Mr. Hodbell’s turn came, he repeated that speech, word by word from memory, though, of course, he couldn’t have seen a line of it before. Now a fine memory is absolutely necessary for this post; and what a memory Mr. Hodbell must have! In addition, Mr. Hodbell did not have his papers in hand, but he was calm. He took the initiative in such a challenging situation, he chose to act and control the situation. This is why we decided that Mr. Hodbell was the exactly the man we wanted.”

As they walked out of the room, the Dean came up to Uncle Theo, who was so confused but so happy that he hardly knew whether he was standing on his head or his heels; and as he shook Theo’s hand he said, “Congratulations, Mr. Hobdell! But, my dear fellow, when you are on our staff, you must be more careful and not leave valuable papers lying about!”

When Uncle Theo came home, there were mass jubilation from his family. A very big and sumptuous party…Uncle Theo and his family were deeply engrossed in their party—totally oblivious to the surroundings.

Exercise

Section 1 Comprehension of the text

1. Read the following statements and decide whether they are true (T) for false (F) according to the text.

1. Adams did not win the post because of his dishonesty. T□ F□

2. When Adams finished his speech, there was hardly any applause. T□ F□

3. Adams knew quite well that Uncle Theo was an absent-minded man. T□ F□

4. Both Uncle Theo and Adams worked day and night at their lectures. T□F□

5. Uncle Theo and Adams presented for different topics. T□ F□

6. Uncle Theo ate like a horse and Adams slept like a log. T□ F□

Section 2 Choose the best answer to each of the following questions based on the information from the text.

1. Which of the following best describes Uncle Theo?

A). Good-mannered B). Modest C). Childish D). Bookish

2. What do we know about the post at Camford University?

A). The applicants had to sit for an examination

B). There was much competition for the post.

C). The post required a lot of teaching experience.

D). The post offered a high salary.

3. Adams did not bother to any preparation because ____

A). He was quite familiar with the subject.

B). He knew the committee members quite well.

C). He had full confidence in himself.

D). He had a well-thought-out plan

4. When Uncle Theo’s turn came, _____

A). He felt so angry that he could not utter a word.

B). He felt so upset that he could not remember anything

C). He had to put the same thoughts in another way

D). He had to repeat the speech, word by word from memory.

5. When the committee went out, Adams_____

A). could not help feeling worried

B). could hardly wait to show his joy

C). felt sorry for Theo and tried to cheer him up

D). felt ashamed and tried to chat with Theo

6. Theo became successful because _____

A). he had a better memory than Adams

B). he was more experienced in teaching than Adams

C). the committee knew he was exactly the man they wanted

D). the committee knew Adams had stolen Theo’s speech.

7. What does the sentence “My uncle Theo is a man with real brains” mean?

A). He has a big brain

B). He is quite positive and lucky

C). He is hard-working and diligent

D). He had the ability to think clearly and learn quickly

Section Comprehension questions. Please use one sentence to answer the questions.

1. How do you describe Uncle Theo?

2. How do you describe Adams?

3. How did Uncle Theo feel when it was his turn to present?

4. What did Uncle Theo present?

5. How did Uncle Theo feel after presentation?

6. How did the dean feel for Uncle Theo’s presentation?

7. Which aspect is the most important one for Uncle Theo to secure the post?

Vocabulary test

Part I. Form recall

Directions: Please supply the English form for a given Chinese word. The first letter of the English word has been provided.

1. J ____________欢呼

2.I____________主动权

3.P____________悲观主义者

4. E ____________爆发

5. C____________胆小

6. I ___________ 威脅

7. S ____________蒙上污名

8. D ____________抨击

9. R ____________报复

10. V ____________摧毁

11. A____________ 好斗的

12. V ____________ 脆弱的

13. D____________严厉的

14. O____________健忘的

15. S ____________奢侈的

Part II. Meaning recall

Direction: Please supply the Chinese translation for the given English word.

1. Jubilation ____________

2.Initiative ____________

3.Pessimist ____________

4. Eruption ____________

5. Cowardice ____________

6. Intimidate ___________

7. Stigmatize ____________

8. Deprecate ____________

9. Retaliate ____________

10. Vandalize ____________

11. Aggressive ____________

12. Vulnerable ____________

13. Draconian ____________

14. Oblivious ____________

15. Sumptuous ____________

Part III Form recognition

Direction: Please choose one option with the meaning that matches the given Chinese word. There are four options for each given Chinese word.

1. 欢呼 A. Lament B. Jubilation C. Reincarnation D. Deduction

2.主动权 A. Sorrow B. Engagement C. Initiative D. Mournfulness

3.悲观主义者 A. Pessimist B. Optimist C. Centralist D. Patriot

4.爆发 A. Eruption B. Sensation C. Turmoil D. Vanquish

5.胆小 A. Audacious B. Cowardice C. Volubility D. Amnesiac

6. 威脅 A. Intimidate B. Conquer C. Annihilate D. Collide

7. 蒙上污名 A. Distress B. Stigmatize C. Strike D. Strengthen

8. 抨击 A. Assent B. Deprecate C. Affirm D. Debilitate

9. 报复 A. Denounce B. Retaliate C. Reticulate D. Denominate

10.摧毁 A. Vandalize B. Vendible C. Venial D. Vegetate

11.好斗的 A. Abolish B. Abandon C. Adhesive D. Aggressive

12.脆弱的 A. Adamant B. Vulnerable C. Viable D. Evitable

13.严厉的 A. Droning B. Draining C. Draconian D. Distraint

14.健忘的 A. Oblivious B. Latency C. Incubate D. Vacuous

15.奢侈的 A. Magnificent B. Sumptuous C. Tenuous D. Sinuous

Part IV Meaning recognition

Direction: Please choose one option with the meaning that matches the given English word. There are four options for each given English word.

1. Jubilation A. 分享 B. 欢呼 C. 欢迎 D. 支撑

2.Initiative A. 主力 B. 主动权 C. 控制欲 D. 粉丝

3.Pessimist A. 残缺 B. 乐观主义 C. 悲观主义 D. 踊跃

4. Eruption A. 报仇 B. 暴乱 C. 爆发 D. 涌动

5. Cowardice A. 雀跃 B. 胆大 C. 动力 D. 胆小

6. Intimidate A. 威脅 B. 冒險 C. 毀壞 D. 嫉妒

7. Stigmatize A. 信任 B. 蒙上污名 C. 专递 D. 强拆

8. Deprecate A. 支持 B. 抨击 C. 违法 D. 遵循

9. Retaliate A. 报复 B. 痛苦 C. 强忍 D. 抱负

10. Vandalize A. 摧毁 B. 催动 C. 催泪 D. 催促

11. Aggressive A. 快乐的 B. 好斗的 C. 欢呼的 D. 有野心的

12. Vulnerable A. 坚强的 B. 乐观的 C. 脆弱的 D. 有后台的

13. Draconian A. 得过且过的 B. 严厉的 C. 忙碌的 D. 魅力的

14. Oblivious A. 健忘的 B. 记忆力好的 C. 忘怀的 D. 难忘的

15. Sumptuous A. 难过的 B. 易燃的 C. 强壮的 D. 奢侈的
